# Proteomic analysis of lysine 2-hydroxyisobutyryl in SLE reveals protein modification alteration in complement and coagulation cascades and platelet activation Pathways

**DOI:** 10.1186/s12920-023-01656-y

**Published:** 2023-10-16

**Authors:** Chaoying Kuang, Dandan Li, Xianqing Zhou, Hua Lin, Ruohan Zhang, Huixuan Xu, Shaoying Huang, Fang Tang, Fanna Liu, Donge Tang, Yong Dai

**Affiliations:** 1 Nephrology Department, The First Affiliated Hospital of Jinan University, Jinan University, Guangzhou, Guangdong, 510632 China; 2 Department of Nephrology, The 924th Hospital of the Chinese People’s Liberation Army Joint Logistic Support Force, Guilin, Guangxi 541002 China; 3 Clinical Medical Research Center, Guangdong Provincial Engineering Research Center of Autoimmune Disease Precision Medicine, Shenzhen Engineering Research Center of Autoimmune Disease, Shenzhen People’s Hospital, The Second Clinical Medical College of Jinan University, Shenzhen, Guangdong 518020 China; 4 Experimental Center, Shenzhen Pingle Orthopedic Hospital (Shenzhen Pingshan Traditional Chinese Medicine Hospital), Shenzhen, Guangdong 518118 China; 5 The First Affiliated Hospital, School of Medicine, Anhui University of Science and Technology, Huainan, Anhui 232001 China

**Keywords:** Systemic lupus erythematosus, Protein post-translational modification, Proteomic, 2-Hydroxyisobutyrylation

## Abstract

**Background:**

Post-translational modifications (PTMs) are considered to be an important factor in the pathogenesis of Systemic lupus erythematosus (SLE). Lysine 2-hydroxyisobutyryl (Khib), as an emerging post-translational modification of proteins, is involved in some important biological metabolic activities. However, there are poor studies on its correlation with diseases, especially SLE.

**Objective:**

We performed quantitative, comparative, and bioinformatic analysis of Khib proteins in Peripheral blood mononuclear cells (PBMCs) of SLE patients and PBMCs of healthy controls. Searching for pathways related to SLE disease progression and exploring the role of Khib in SLE.

**Methods:**

Khib levels in SLE patients and healthy controls were compared based on liquid chromatography tandem mass spectrometry, then proteomic analysis was conducted.

**Results:**

Compared with healthy controls, Khib in SLE patients was up-regulated at 865 sites of 416 proteins and down-regulated at 630 sites of 349 proteins. The site abundance, distribution and function of Khib protein were investigated further. Bioinformatics analysis showed that Complement and coagulation cascades and Platelet activation in immune-related pathways were significantly enriched, suggesting that differentially modified proteins among them may affect SLE.

**Conclusion:**

Khib in PBMCs of SLE patients was significantly up- or down-regulated compared with healthy controls. Khib modification of key proteins in the Complement and coagulation cascades and Platelet activation pathways affects platelet activation and aggregation, coagulation functions in SLE patients. This result provides a new direction for the possible significance of Khib in the pathogenesis of SLE patients.

**Supplementary Information:**

The online version contains supplementary material available at 10.1186/s12920-023-01656-y.

## Introduction

Systemic lupus erythematosus (SLE) is an autoimmune disease, the global SLE incidence and prevalence are estimated to be 5.14 per 100,000 person-years and 43.7 per 100,000 persons for the overall population [1]. However, its epidemiology varies considerably worldwide and is influenced by factors such as geographic region, age and sex of the population [1]. SLE causes the termination and destruction of immune tolerance, resulting in the production of a large number of autoantibodies, which eventually causes the skin, kidneys, heart, joints and other organs damage. The pathogenesis of SLE is complex and has not been fully elucidated. It is generally believed that genetic, immune and environmental factors are involved in its pathogenesis [2, 3]. With the development of sequencing technology and bioinformatics technology, and the introduction of epigenetics, genomics, transcriptomics, and proteomics, the pathogenesis of SLE has been recognized and analyzed at multiple levels. Among them, post-translational modification (PTM) has been regarded as an important factor in the pathogenesis of SLE in recent years [4]. PTM of proteins refers to the covalent modification of proteins produced by gene expression. Generally, the translated protein precursor is inactive. To become a protein with a certain function, it needs to undergo post-translational processing. This process is PTM. Some PTMs cause changes in protein structure that affect protein properties and function. PTM is essential for the occurrence and development of many biological processes and diseases. Ubiquitination, succinylation, acetylation, methylation, and crotonylation are the common types of PTM [5–9]. In SLE, there are many related studies demonstrating the role of PTM in the pathogenesis of SLE. Human cathelicidin is an antimicrobial peptide, and LL37 corresponds to the COOH terminal part of the molecule [10]. The study [6, 11] by R. Lande et al. found that citrullinated post-translational modifications of LL37 were readily detected in SLE-affected target organs (skin and kidney), and that cit-LL37 (citrullinated LL37) may have further enhanced T cell response [6, 11]. Another of their studies detected carbamylated LL37 in tissues affected by SLE [12]. Anti-natural LL37 antibodies have potential pathogenic effects in SLE, and this carbamylated LL37 can promote the production of anti-natural LL37 antibodies through activation of T helper cells and direct effects on B cell differentiation [12].

Lysine 2-hydroxyisobutyrylation (Khib), a PTM discovered in 2014, can cause significant structural changes to proteins [13]. Khib is widely involved in a variety of important physiological processes in animals and plants, with effects on biological metabolic activities, cell lifespan regulatory networks, and more [14, 15]. Autoimmune diseases are closely related to this PTM. The P13K-Akt signaling pathway in psoriatic skin lesions is significantly up-regulated by Khib protein modification, and HSP90 and 14-3-3 proteins on this pathway are significantly up-regulated by khib, which may ultimately affect psoriatic keratinization and cellularity proliferation rate [16]. Khib protein is enriched in the IL-17 signaling pathway and phagosome class in IgA nephropathy and may be involved in the pathogenesis of IgA nephropathy [17]. SLE is a common systemic autoimmune disease with multiple organ and tissue involvement, but little is known about the biochemical function of Khib in SLE patients.

## Methods

### Selection of research object and sample preparation

**Fig. 1:**
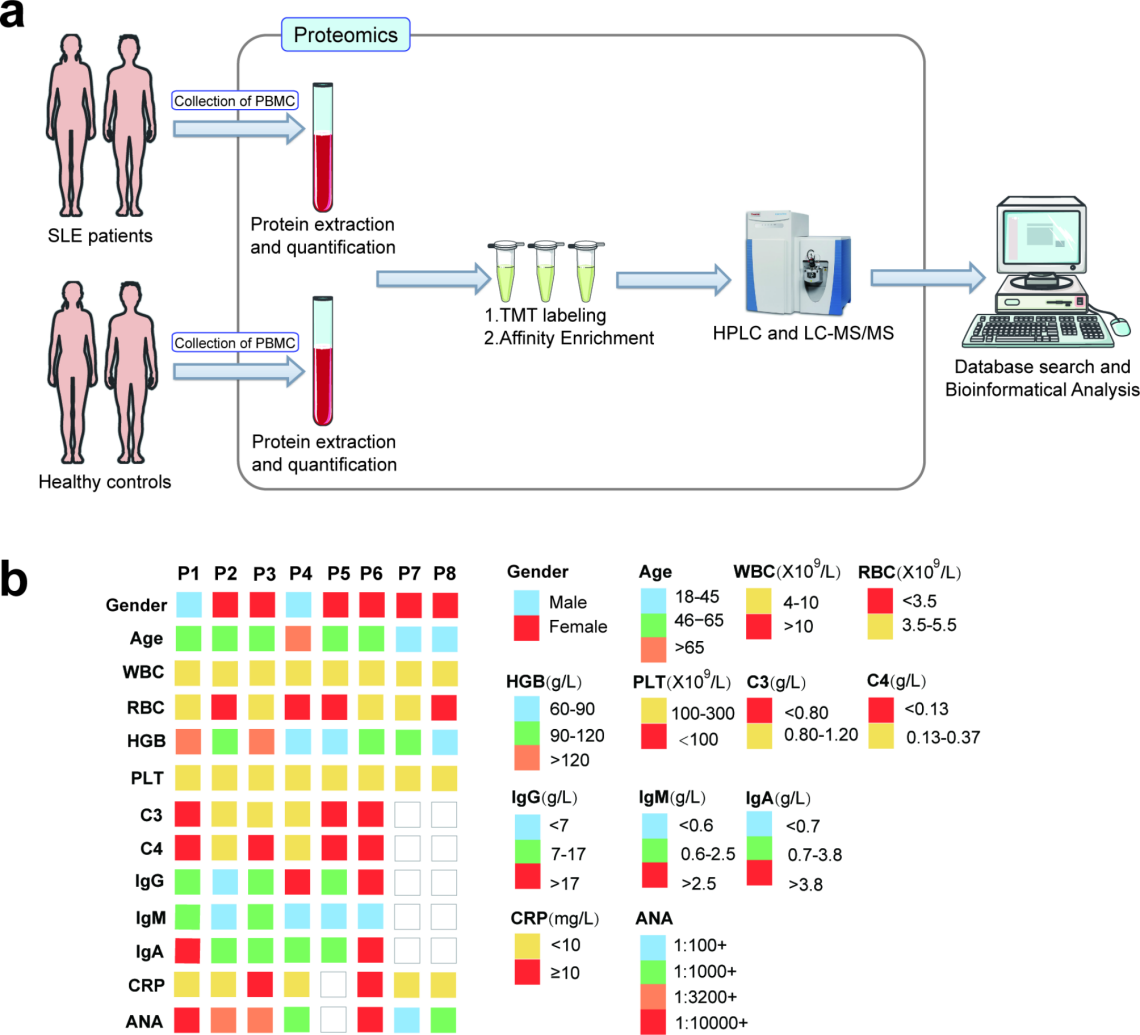
Sample information and experimental process. **(a)** Sample preparation and bioinformatics workflow. This image was drawn by Adobe Illustrator 2019, and the materials are from SERVER MEDICAL ART (https://smart.servier.com). **(b)** Clinical information for SLE patients

Under the guidance of the program approved by the Guangxi Key Laboratory of Metabolic Diseases Research Ethics Committee, we collected Peripheral blood mononuclear cells (PBMCs) samples from 8 SLE patients [18]. All patients had no other obvious complications or serious primary diseases, such as cardiovascular disease, liver disease, and so on. In addition, 8 normal controls were screened, and they did not have any other diseases through health examination. This study was approved by Guangxi Key Laboratory of Metabolic Diseases Research Ethics Committee, and each participant signed an informed consent form. The clinical parameters of SLE patients are shown in Fig. 1b.

After adding the buffer solution (8 M urea and 1% protease inhibitor cocktail) to the peripheral blood sample, the extracted protein was obtained by ultrasonic lysis, centrifugation, and removal of cell debris, followed by determination of protein concentration using a BCA kit. To the prepared protein solution, trypsin was added twice and lysed, and the peptides obtained after lysis were labeled with TMT reagent. The whole proteolytic peptides were separated by High Performance Liquid Chromatography (HPLC). In order to enrich 2-hydroxyisobutyryl modified peptide, the supernatant of the trypsin peptide dissolved in NETN buffer (100 mM NaCl, 1 mM EDTA, 50 mM Tris-HCl, 0.5% NP-40, pH 8.0) was transferred to a 2-hydroxyisobutyrylated resin (Lot number PTM-804, PTM Bio Inc, Hangzhou). After incubation at 4 °C with gentle shaking overnight, the beads were washed with NETN buffer and H_2_O, respectively. The bound peptides were then eluted from the magnetic beads with 0.1% trifluoroacetic acid, desalted with C18 ZipTips (Millipore), and dried by a vacuum spin dryer. Next, the peptide was analyzed by LC-MS/MS, and the specific protein information corresponding to the mass spectrum data was retrieved in Maxquant (v1.5.2.8), followed by bioinformatics analysis. Please see the Supporting Information for the detailed methods.

### Bioinformatics analysis

For experimentally derived data, SLE/NC ratios ≥ 1.2 or ≤ 1/1.2 were considered differentially expressed. Venn diagrams were drawn on the ehbio website (http://www.ehbio.com/test/venn/). Subcellular localization annotation of proteins using wolfpsort. Gene Ontology (GO) analysis was performed using the DAVID website (http://david.ncifcrf.gov), and then GO bubble maps were drawn on the Oebiotech website (https://cloud.oebiotech.cn/task/). KEGG analysis was performed by Cytoscape (v3.8.2) software. The original KEGG pathway map was downloaded from the Kyoto Encyclopedia of Genes and Genomes (KEGG) (https://www.genome.jp/kegg/). On the basis of the original pathway map, we use the materials from SERVER MEDICAL ART (https://smart.servier.com) to adjust and process the KEGG pathway map. The STRING website (https://cn.string-db.org) was used to analyze the interaction between proteins, and the Cytoscape (v3.8.2) software was used to draw the PPI network diagram. The MCODE plug-in in Cytoscape was used to extract the sub-network, and the KEGG pathway enrichment analysis was carried out on the differentially modified proteins (DMPs) in the sub-network. More detailed experimental procedures are in Supplementary Materials.

## Results

We performed the experimental procedure in 4 major steps (Fig. 1a). Firstly, PBMCs samples from SLE patients and healthy controls were collected. After the protein is extracted, TMT labeling, HPLC grading, and LC–MS/MS (for PTM experiment, Khib antibody affinity enrichment is also required before this step) are used for protein sequencing and PTM experiment. Then, comprehensive methods such as database search and bioinformatics analysis are used for analysis to explore the role of Khib in SLE.

The clinical information of 8 SLE patients is shown in Fig. 1b. Most of the patients are between 45 and 65 years old. All patients had no thrombocytopenia (< 100 × 109/L) and leukopenia (< 3 × 109/L), and ANA was positive. RBC of 4 patients was lower than normal (< 3.5 × 109/L). Most patients have mild to moderate anemia. Complement C3 decreased in 3 patients (< 0.80 g/L) and C4 decreased in 4 patients (< 0.13 g/L). Among immunoglobulins, IgG decreased in 1 patient (< 7 g/L) and increased (> 17 g/L) in 2 patients. IgM decreased in 2 patients (< 0.6 g/L). IgA increased in 2 patients (> 3.8 g/L). CRP increased in 2 patients (≥ 10 mg/L).

In studies related to the pathogenesis of SLE, dysregulation of RNA processing has been shown to play a crucial role in the pathogenesis of SLE. It has been shown that the binding of immune complexes containing DNA, RNA or RNA-binding proteins to plasmacytoid dendritic cells triggers the overproduction of αIFN in SLE patients [19], which in turn causes activation of self-reactive lymphocytes, dysfunction of regulatory T cells, and dysregulation of endothelial cells and angiogenesis [20–22]. In mRNA, any disruption or mutation in the mRNA splicing machinery may affect the production of mature mRNA and functional proteins, with implications for the pathogenesis of SLE.

**Fig. 2:**
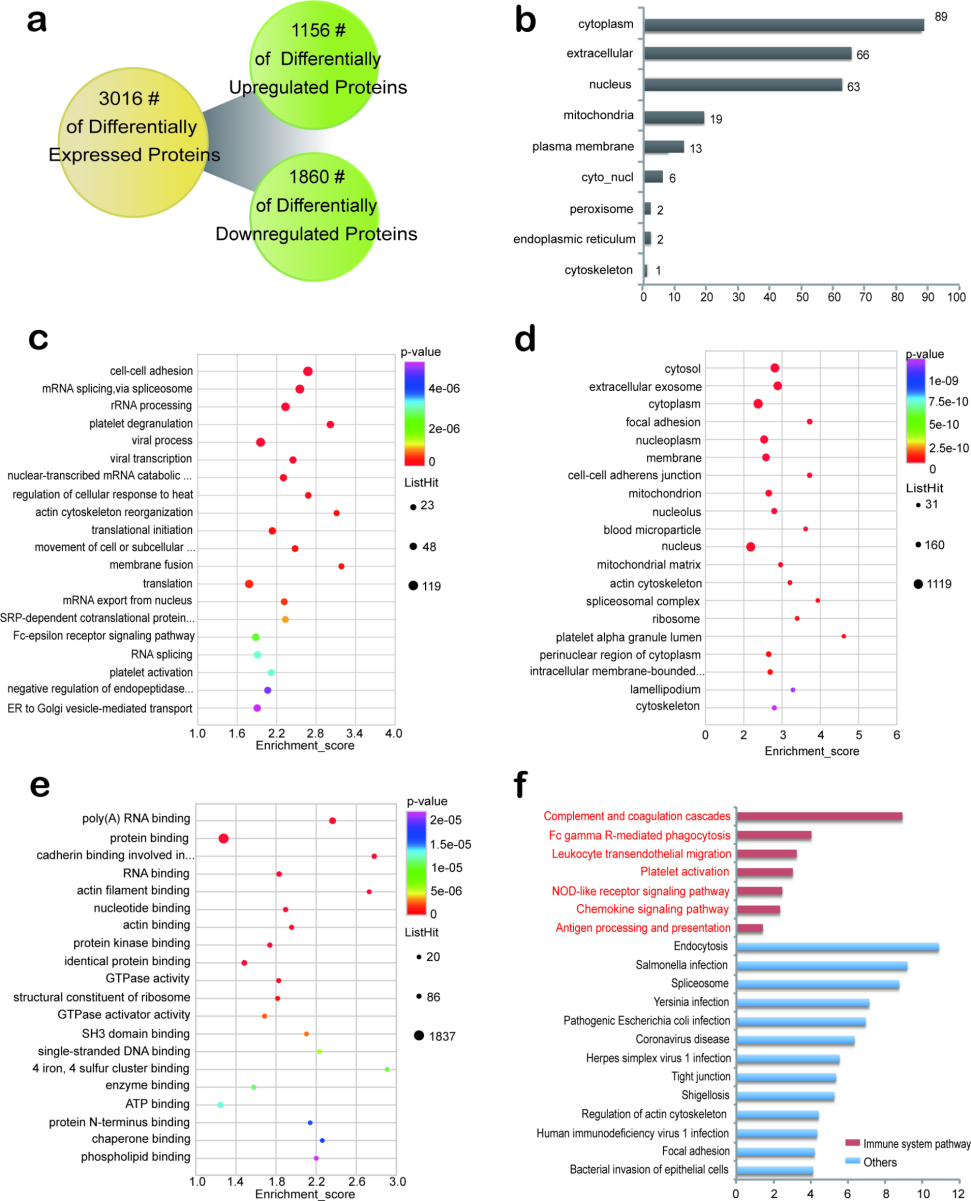
Quantitative analysis, GO enrichment analysis and KEGG enrichment analysis of 2-hydroxyisobutyrylated differentially expressed proteins in SLE patients. **(a)** Quantification of differentially expressed proteins in a Venn diagram. **(b)** Subcellular localization of differentially expressed proteins. **(c)** Biological processes in GO enrichment analysis. **(d)** Cellular components in GO enrichment analysis. **(e)** Molecular functions in GO enrichment analysis. **(f)** KEGG analysis of differentially expressed proteins

### Quantification, functional properties and functional enrichment of differentially expressed proteins in PBMCs of SLE patients

Differential expression criteria for differentially expressed proteins and DMPs were as follows: fold change > 1.2 was considered up-regulated, and < 1/1.2 was considered down-regulated. A total of 3016 differentially expressed proteins (DEPs) were identified, of which 1156 were up-regulated and 1860 were down-regulated (Fig. 2a). The subcellular localization of DEPs is mainly distributed in cytoplasm (34.1%), extracellular (25.3%), nucleus (24.1%), mitochondria (8.3%), and plasma membrane (5.0%) (Fig. 2b). The GO enrichment analysis of DEPs shows that in the biological process category, proteins are mainly enriched in biological process terms such as cell-cell adhesion, mRNA splicing, via spliceosome, rRNA processing, platelet degranulation and RNA splicing (Fig. 2c). In the cell composition category of GO, DEPs are mainly enriched in cytosol, extracellular exosome and cytoplasm (Fig. 2d). Among the molecular functional categories, DEPs were mainly enriched in poly(A) RNA binding, protein binding, cadherin binding involved in cell-cell adhesion, and RNA binding (Fig. 2e). Analysis of KEGG enrichment revealed that DEPs were associated with multiple immune pathways, including Complement and coagulation cascades, and Platelet activation (Fig. 2f).

### Quantitative, functional characterization of DMPs in PBMCs of SLE patients

**Fig. 3:**
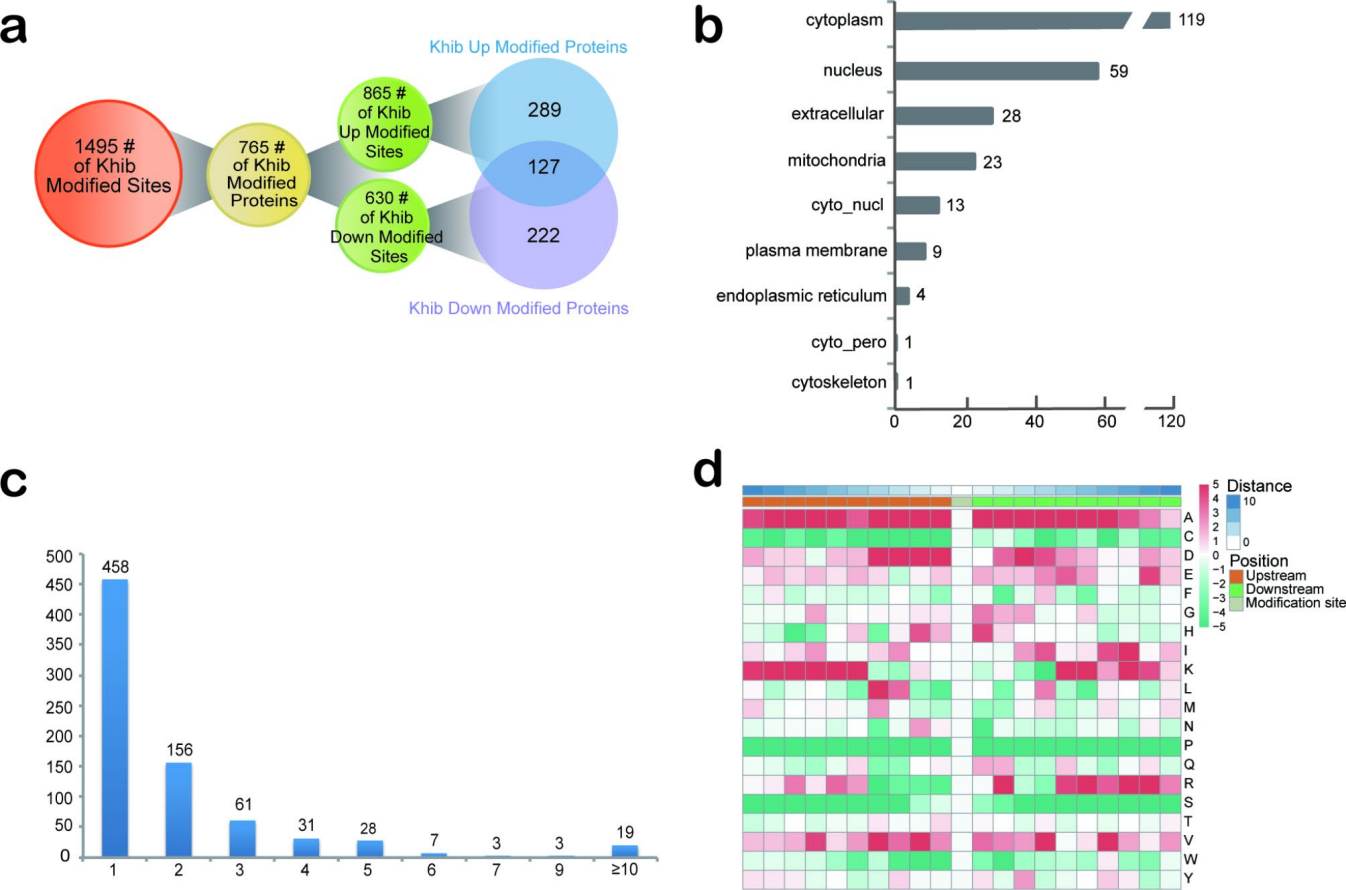
Quantitative and qualitative analysis of 2-hydroxyisobutyrylated differentially modified proteins (DMPs) in SLE patients. **(a)** Quantification of Khib DMPs in a Venn diagram. **(b)** Subcellular localization of Khib DMPs. **(c)** Distribution of the number of Khib sites per protein. **(d)** Heatmaps of Khib DMPs. It shows the frequency of modifying some amino acids around lysine. Red indicates high frequency and green indicates low frequency

In the differential modified protein group, 1495 Khib differentially modified sites of 765 Khib DMPs were identified. 856 sites were differentially modified in 416 up-regulated DMPs, and 630 sites were differentially modified in 349 down-regulated DMPs. Among all DMPs, 127 proteins have both up-regulated and down-regulated differentially modified sites (Fig. 3a). The subcellular localization of Khib DMPs is mainly distributed in the cytoplasm (46.3%), nucleus (23.0%), extracellular (10.9%), mitochondria (8.9%), cyto_nucl (5.1%) (Fig. 3b). Furthermore, the distribution number of modification sites in each Khib DMP was determined, of which 458 proteins contained only one Khib site (Fig. 3c).

The heatmap of the amino acid sequence around the 2-hydroxyisobutyryl site shows that some amino acid residues around Khib are significantly enriched (Fig. 3d). Residue A is enriched at positions − 10 to − 1 and 1 to 9, while residues D, K and R are significantly enriched at positions − 4 to − 1 and 2 to 4, − 10 to − 5 and 5 to 6 and 8 to 9, 5 to 9, respectively.

### Functional enrichment of up-regulated Khib DMPs in PBMCs of SLE patients

**Fig. 4:**
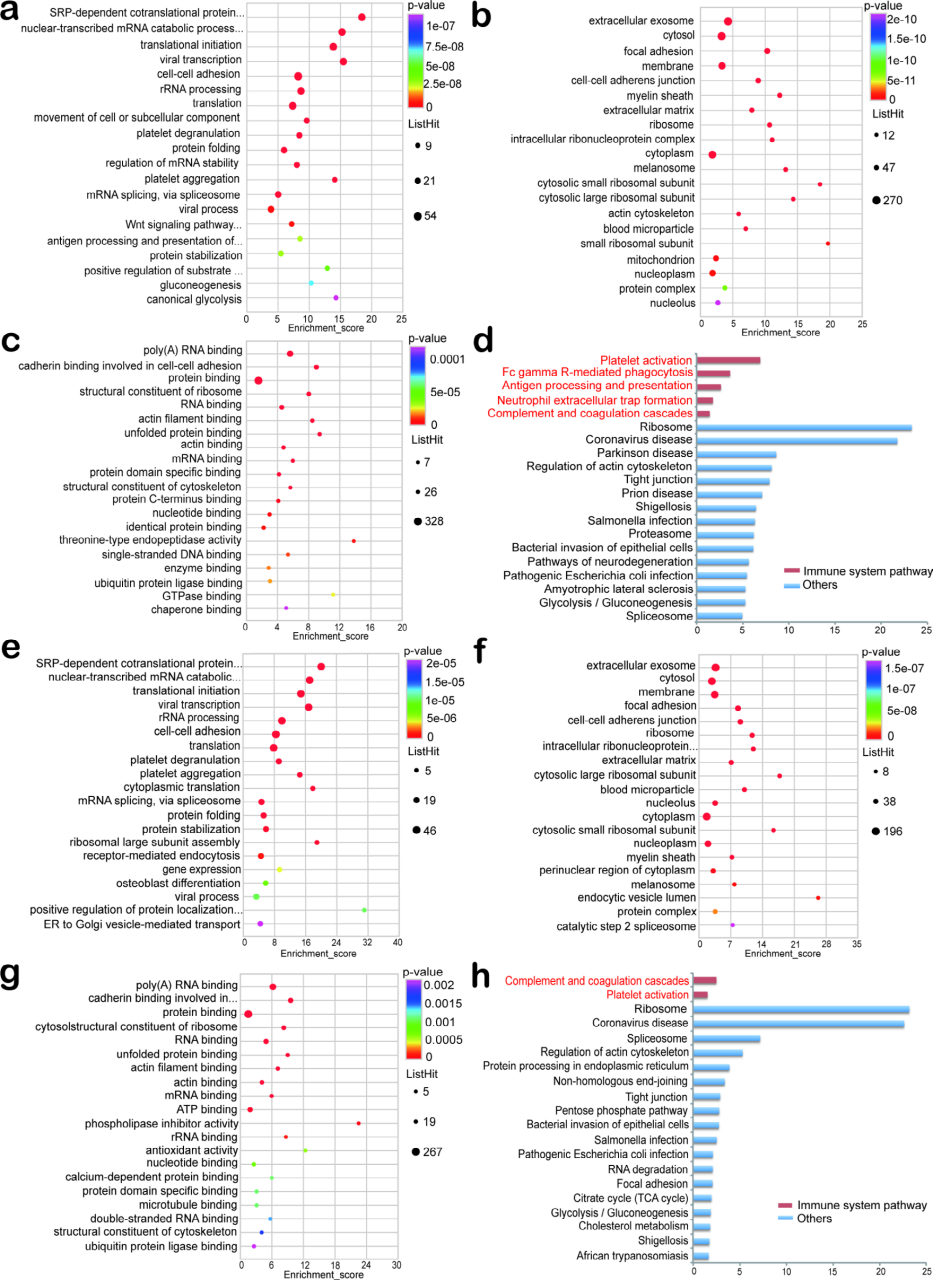
GO enrichment analysis and KEGG pathway enrichment analysis of Khib DMPs. **(a-d)** Biological process, cell composition, molecular function in GO enrichment analysis of up-regulated Khib DMPs, and KEGG pathway enrichment analysis of up-regulated Khib DMPs. **(e-h)** Biological process, cell composition, molecular function in GO enrichment analysis of down-regulated Khib DMPs, and KEGG pathway enrichment analysis of down-regulated Khib DMPs

To understand which functions are mainly affected by Khib DMPs in PBMCs of SLE patients, we analyzed the identified data with GO. The results showed that in biological processes (Fig. 4a), up-regulated Khib DMPs were mainly enriched in SRP-dependent cotranslational protein targeting to membrane, nuclear-transcribed mRNA catabolic process, nonsense-mediated decay, translational initiation and mRNA splicing, via spliceosome. In the cellular composition category (Fig. 4b), up-regulated Khib DMPs were mainly enriched in extracellular exosome, cytosol, and focal adhesion. Among the molecular functions (Fig. 4c), up-regulated Khib DMPs were mainly enriched for poly(A) RNA binding, cadherin binding involved in cell-cell adhesion, protein binding, RNA binding, and mRNA binding. Analysis of KEGG enrichment revealed that up-regulation of Khib DMPs was associated with multiple immune pathways, including Complement and coagulation cascades, and Platelet activation (Fig. 4d).

### Functional enrichment of down-regulated Khib DMPs in PBMCs of SLE patients

The results of GO enrichment analysis showed that in biological processes (Fig. 4e), down-regulated Khib DMPs were mainly enriched in SRP-dependent cotranslational protein targeting to membrane, nuclear-transcribed mRNA catabolic process, nonsense-mediated decay, translational initiation, and mRNA splicing, via spliceosome. In the cellular composition category (Fig. 4f), down-regulated Khib DMPs were mainly enriched in extracellular exosome, cytosol, and membrane. Among the molecular functions (Fig. 4g), down-regulated Khib DMPs were mainly enriched in poly(A) RNA binding, cadherin binding involved in cell-cell adhesion, protein binding, RNA binding, and mRNA binding. Analysis of KEGG enrichment showed that down-regulated Khib DMPs were significantly enriched in immune-related pathways that Complement and coagulation cascades, and Platelet activation (Fig. 4h). In the results of our bioinformatics analysis (Figs. 2 and 4), many RNA-related alterations such as RNA splicing, mRNA splicing, RNA binding, mRNA binding can be seen.These results also further confirm that the dysregulation of RNA processing described above plays a crucial role in the pathogenesis of SLE.

### Analysis of functional modules of Khib DMPs interaction network

**Fig. 5:**
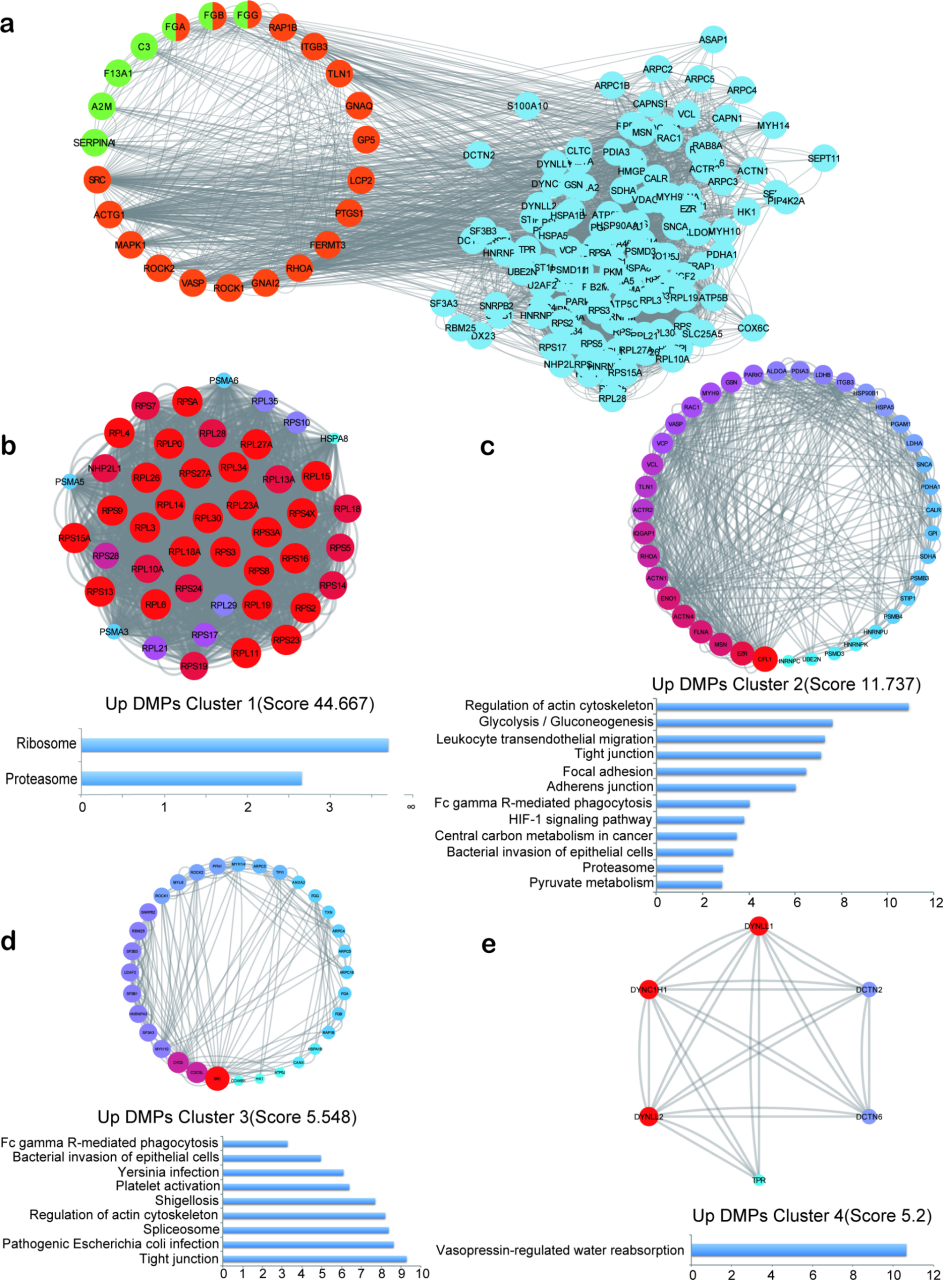
PPI network analysis of up-regulated Khib DMPs. **(a)**The green color is DMPs in Complement and coagulation cascades, and the orange color is DMPs in Platelet activation. Use the MCODE plug-in to extract the first four clusters and perform KEGG analysis on them **(b, c, d, e)**

**Fig. 6:**
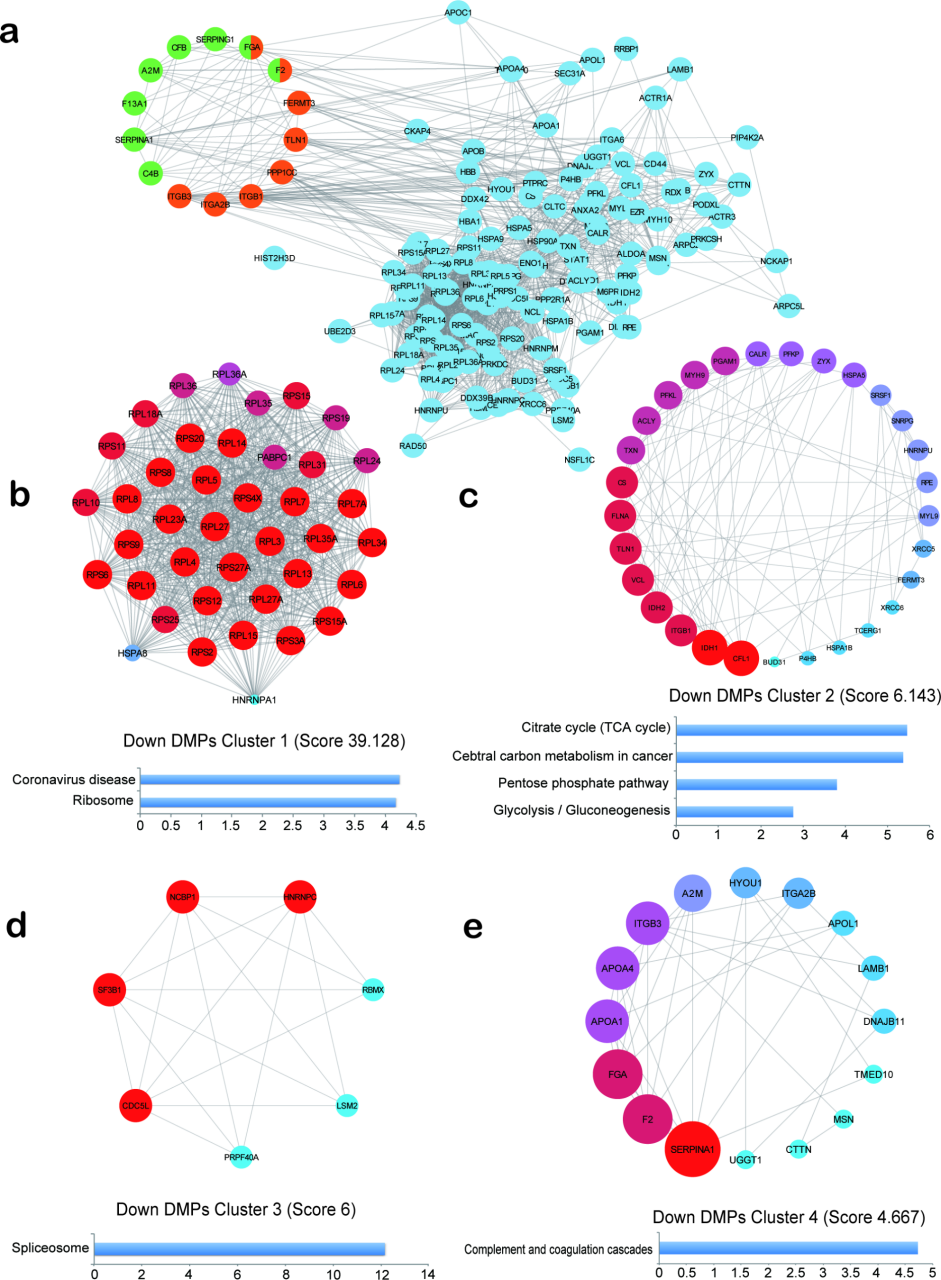
PPI network analysis of down-regulated Khib DMPs. **(a)** DMPs in the Complement and coagulation cascades are shown in green, and those in Platelet activation are shown in orange. **(b, c, d, e)** Use the MCODE plug-in to extract the first four clusters and perform KEGG analysis on them

In order to gain a deeper understanding of the biological functions of Khib DMPs, we selected DMPs in all pathways in Khib DMPs’ KEGG maps (Fig. 4d, h), and constructed PPI maps (Figs. 5a and 6a) through STRING database and Cytoscape (v3.8.2). The first four clusters were extracted from the PPI network with Khib DMPs upregulated by MCODE plug-in, and the KEGG pathway enrichment analysis was carried out for the DMPs among them. Cluster1 (Score 44.667) is mainly enriched in Ribosome and Proteasome (Fig. 5b). Cluster 2 (Score 11.737) is mainly enriched in Regulation of actin cytoskeleton, Glycolysis/Gluconeogenesis, Leukocyte transendothelial migration (Fig. 5c). Cluster 3 (Score 5.548) is mainly enriched in Fc gamma R-mediated phagocytosis, Bacterial invasion of epithelial cells, Yersinia infection and Platelet activation (Fig. 5d). Cluster 4 (Score 5.2) is mainly enriched in Vasopressin-regulated water reabsorption (Fig. 5e).

Similarly, MCODE also extracts the first four clusters from the PPI network that downregulates Khib DMPs. Cluster1 (Score 39.128) is mainly enriched in Coronavirus disease and Ribosome (Fig. 6b). Cluster2 (Score 6.143) is mainly enriched in the Citrate cycle (TCA cycle), Cebtral carbon metabolism in cancer, and Pentose phosphate pathway (Fig. 6c). Cluster 3 (Score 6) is mainly enriched in Spliceosome (Fig. 6d). Cluster 4 (Score 4.667) is mainly enriched in Complement and Coagulation Cascades (Fig. 6e).

The above further indicates that Khib may play a role in immune-related pathways.

### Characterization of complement and coagulation cascades and platelet activation pathways

**Fig. 7:**
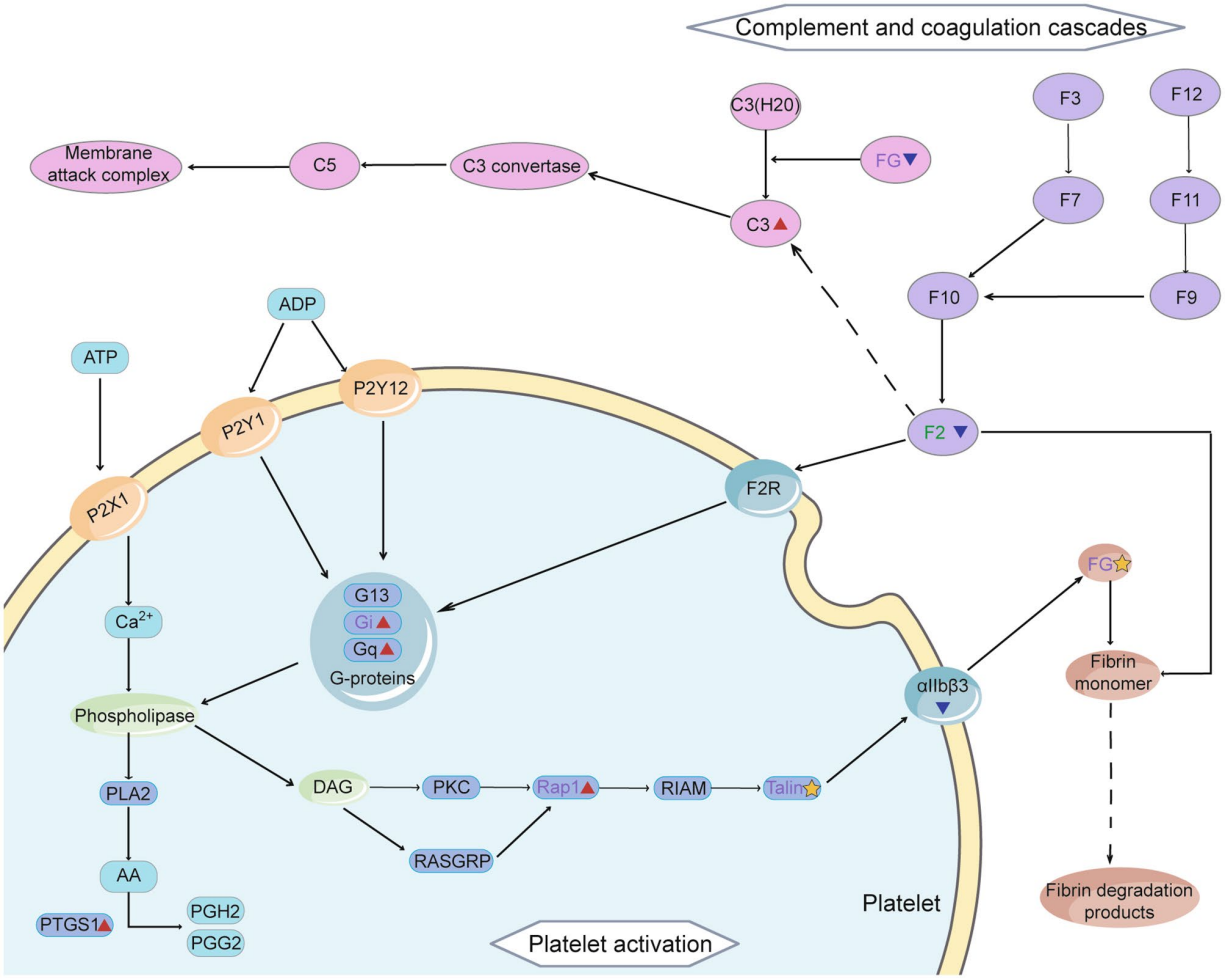
Complement and coagulation cascades and Platelet activation pathways. Red triangles represent up-regulated Khib DMPs, blue triangles represent down-regulated Khib DMPs, and yellow stars represent Khib DMPs with both up- and down-regulated sites. Green fonts indicate up-regulated differentially expressed proteins, and purple fonts indicate down-regulated differentially expressed proteins. This image was drawn by Adobe Illustrator2019, and the materials are from SERVER MEDICAL ART (https://smart.servier.com)

11 Khib DMPs were significantly enriched in the Complement and coagulation cascades pathway, including A2M, C3, C4B, FGA, FGB, FGG, F13, F2, SERPINA1, CFB, SERPING1 (Table S1). 23 Khib DMPs on the Platelet activation pathway were significantly enriched, including FGA, FGB, FGG, F2, PTGS1, RhoA, Rock1, Rock2, PPPIC, GNAQ, GNAI2, RAP1A, VASP, ACTG1, FERMT3, TLN1, TIGA2B, ITGB3, SRC, ITGB1, LCP2, GP5, MAPK1 (Table S1). SLE is an immune-related disease, and complement, coagulation, and platelet activation are closely related to immunity. These two pathways are the focus of our attention, as Fig. 7 illustrates.

## Discussion

In recent years, more and more studies have proved that there is a close relationship between PTM and SLE. Our study explored the role of Khib in SLE. In this study, through proteomic analysis of PBMCs of SLE patients and healthy controls, we found that Khib in PBMCs of SLE patients and healthy controls were significantly different. It is generally believed that SLE is an immune-related disease. The deposition of its autoimmune complex in local tissues will cause inflammatory reactions and tissue damage [24]. As immune-related pathways, Complement and Coagulation cascades and Platelet activation are significantly enriched in KEGG analysis. Therefore, we further studied the two pathways that may affect SLE.

We observed that the subcellular localization of Khib DMPs was mainly distributed in the cytoplasm and nucleus, and only a few proteins were annotated in other parts such as mitochondria. This result suggests that Khib may have an important influence on cell structure and important biological processes in cells. GO enrichment analysis showed that protein Khib was significantly enriched on immune and platelet-related terms. It further indicates that Khib may be closely related to coagulation and immunity.

In our other paper on SLE [18], we found that co-modification of lysine crotonylation (Kcr) and Khib occurring in HSPA8, a key protein in the Antigen processing and presentation pathway, may increase ATP hydrolysis and promote antigen binding to MHC II molecules, which may be associated with the pathogenesis of SLE. In our enrichment analysis of the KEGG pathway, several immune-related pathways were identified with significant enrichment, and the Antigen processing and presentation pathway was among them. This showed that Khib, when modified independently of Kcr, may also have an impact on the pathogenesis of SLE through proteins on this pathway. In Addition to this, we noted Complement and coagulation cascades as well as Platelet activation pathways. Both pathways were enriched in the KEGG analysis of both differentially expressed and differentially modified proteins, as well as in the results of the protein interaction network analysis. The complement system and coagulation cascade are important components of the immune system, and they regulate and influence each other with inflammatory factors as a bridge [23]. In SLE, the circulating immune complex activates complement, leading to the formation of complement fragments [24]. Platelet binding complement fission products may induce downstream immune-mediated platelet activation, leading to enhanced platelet aggregation after stimulation [25]. In conclusion, the relationship between complement, coagulation, platelet activation, and their relationship with SLE is very close. Given their close relationship, if the key proteins in the Complement and coagulation cascades pathway and the Platelet activation pathway are abnormally modified in SLE patients, platelet activation, coagulation and other functions are likely to be affected. Therefore, we further investigated the Complement and coagulation cascades and Platelet activation pathways.

In our study, 11 Khib DMPs were enriched in the Complement and coagulation cascades pathway, including FGA, FGB, FGG. And 23 Khib DMPs were enriched in the Platelet activation pathway, including FGA, FGB, FGG, PTGS1. Due to the presence of lupus anticoagulants and circulating immune complexes, SLE patients often have coagulation and fibrinolysis disorders, most patients have thrombosis, including large blood vessels, small blood vessels, and Libmin-Sacks endocarditis, and a small number of patients may have an obvious bleeding tendency [26, 27].

As an indicator of thrombotic status, fibrinogen is an important component of the Complement and coagulation cascades and the Platelet activation pathway. The fibrinogen molecule is a symmetrical dimer composed of three pairs of different polypeptide chains, Aα, Bβ, and γ, connected by disulfide bonds [28]. These three pairs of peptide chains are encoded by three independent genes, FGA, FGB, and FGG, respectively. Many studies have shown that many PTMs on different parts of the fibrinogen peptide chain can produce different clot structures [29, 30], and some PTMs can cause conformational changes in fibrinogen, which affect the stability of the thrombus [30, 31]. Almost all fibrinogen abnormalities affect fibrin polymerization [32]. The αC region is consisted by αC domain (Aα392–610) and αC connector (Aα221–391), located at the C-terminus of the fibrinogen Aα chain [33]. The N-segment subdomain of the αC region (Aα441–496) has a double hairpin structure with dynamic features, that is, open or closed. Various hairpin-hairpin interactions occur in multiple orientations of the hairpin in a closed conformation of the hairpin [34]. The hairpin allows support from multiple directions, including charged amino acids in the hairpin region of this peptide [34]. These amino acids combine with other side chains through hydrogen bonds to form salt bridges and combine with oxygen atoms and nitrogen atoms of the main chain [34]. We identified multiple khib sites on FGA, FGB and FGC, among which K558, K620, K581, K448, K463, K476, K575 and K599 were located in the αC region of Aα chain. Three Khib sites (K448, k463, K476) were located in the hairpin structure of the αC region and were down-regulated. Unmodified lysines are positively charged and cannot form hydrogen bonds and bind to other side chains. Khib reverses the original positive charge of lysine to a negative charge, enabling lysine to form hydrogen bonds and bind to other side chains, providing support for the stability of the closed conformation hairpin. However, in SLE patients, the Khib site in the N-segment subdomain of the αC region was significantly down-regulated, implying a relatively reduced support for hairpin stability, possibly causing conformational changes in fibrinogen and affecting fibrin polymerization. Platelet aggregation may also be affected by abnormal fibrinogen production [35].

Immune thrombocytopenia is one of the most common hematologic manifestations of SLE. Most SLE patients have a hypercoagulable state and a tendency to thrombosis, and some have a bleeding tendency. Prostaglandin endoperoxide synthase 1 (PTGS1) is an important component of the Platelet activation pathway. Prostaglandin synthases (PTGSs), also known as cyclooxygenase enzymes (COXs), exist in two isoforms: PTGS1 (also known as COX1) and PTGS2 (also known as COX2) [36]. The conversion of arachidonic acid to prostaglandin H2 (PGH2) is mediated by 2 different isozymes: constitutive PTGS1 and inducible PTGS2 [37]. PTGS1 has peroxidase activity, which can first oxidize arachidonic acid to PGG2, and then reduce it to hydroxy endoperoxide PGH2 [38]. In platelets, PGH2 is involved in the production of thromboxane A2 (TXA2), which promotes platelet activation and aggregation, and vasoconstriction [39]. The major domains of PTGS1 include the epidermal growth factor domain (sites 34–72), the membrane-binding domain (sites 73–116), and the catalytic domain (sites 117–586) [40]. The COX-1 active site contains a hydrophobic inverted L-shaped channel that penetrates deep into the catalytic domain above the membrane-bound domain [40]. The four helices of the membrane-binding domain surround to form a space in which Ser-530 is located [40]. Aspirin mainly irreversibly acetylates the hydroxyl group of Ser-530 at the active site of PTGS1, and blocking the hydrophobic channel due to the steric hindrance, which will prevent arachidonic acid from entering the active site of the enzyme, hinder the formation of PGG2, affect the synthesis of thromboxane A2, and then exert its anti-platelet aggregation effect [41]. Our study found that PTGS1 on the Platelet activation pathway was modified by Khib at K565 and was significantly up-regulated. K565 is located in the catalytic domain, and Khib lengthens the side chain of the original lysine, which may make the space of the inverted L-shaped channel smaller, affecting the entry of arachidonic acid from the hydrophobic channel and binding to the active site. The production of PGH2 is thus affected, inhibition of platelet aggregation occurs, and the balance mechanism of procoagulation and anticoagulation is disrupted, resulting in an increased risk of bleeding in patients with SLE.

Definitely, our study has some limitations. The study focused on the overall characteristics of Khib in SLE, involving a small number of SLE patients, and did not consider subgroups of SLE patients based on disease activity, severity, and organs involved. Collectively, we demonstrated the presence of abundant Khib DMPs in PBMCs of SLE patients and enrichment in Complement and coagulation cascades and Platelet activation pathways. The possible effects of Khib DMPs on these two pathways in SLE disease were emphatically discussed. The changes in protein structure and function caused by Khib in SLE deserve further exploration and deepen the understanding of the pathogenesis of SLE.

## Conclusion

This study explored the effect of Khib on protein function in PBMCs of SLE patients from the perspective of proteomics. Our study shows that Khib occurring in SLE is associated with multiple immune-related signaling pathways, especially Complement and coagulation cascades and Platelet activation. FGA and PTGS1 on these two pathways have attracted attention. These results suggested that the Khib modification of these key proteins may be related to platelet activation and aggregation, coagulation and other functions in SLE patients, which may provide a new understanding of Khib’s involvement in protein regulation and the disease mechanism of SLE.

### Electronic supplementary material

Below is the link to the electronic supplementary material.

Supplementary Material 1

## Data Availability

The mass spectrometry proteomics data have been deposited to the ProteomeXchange Consortium via the PRIDE [42] partner repository with the dataset identifier PXD040218 (https://www.ebi.ac.uk/pride/profile/reviewer_pxd040218). Username: reviewer_pxd040218@ebi.ac.uk, Password: D4TG3NFq.

## List of abbreviations

COXs: Cyclooxygenase enzymes
DEPs: Differentially expressed proteins
DMPs: Differentially modified proteins
GO: Gene Ontology
HPLC: High Performance Liquid Chromatography
Kcr: Lysine crotonylation
KEGG: Kyoto Encyclopedia of Genes and Genomes
Khib: Lysine 2-hydroxyisobutyrylation
PBMCs: Peripheral blood mononuclear cells
PGH2: Prostaglandin H2
PTGSs: Prostaglandin synthases
PTGS1: Prostaglandin endoperoxide synthase 1
PTM: Post-translational modification
SLE: Systemic lupus erythematosus
TXA2: Thromboxane A2
