# Supplementary material for: Proteomic analysis of lysine 2-hydroxyisobutyryl in SLE reveals protein modification alteration in complement and coagulation cascades and platelet activation Pathways
Source: BMC Med Genomics. 2023 Oct 16;16:247. doi: 10.1186/s12920-023-01656-y (PMC10577913; doi:10.1186/s12920-023-01656-y)
Supplement: Supplementary file 1 — Supplementary Material 1 [file 12920_2023_1656_MOESM1_ESM.docx]

**Supplementary Information**

Proteomic analysis of lysine 2-hydroxyisobutyryl in SLE reveals protein modification alteration in Complement and coagulation cascades and Platelet activation pathways

Chaoying Kuang^1,2#^, Dandan Li^3#^, Xianqing Zhou^2^, Hua Lin^2^, Ruohan Zhang^2^, Huixuan Xu^3^, Shaoying Huang^3^, Fang Tang^2^, Fanna Liu^1^*, Donge Tang^3^*, Yong Dai^1,3^ *

# These authors contributed equally to this study.

1. Institute of Nephrology and Blood Purification, The First Affiliated Hospital of Jinan University, Jinan University, Guangzhou 510632, China
2. Guangxi Key Laboratory of Metabolic Diseases Research, No.924 Hosptital of PLA Joint Logistic Support Force, Guilin, (Guangxi), China.
3. Clinical Medical Research Center, Guangdong Provincial Engineering Research Center of Autoimmune Disease Precision Medicine, Shenzhen Engineering Research Center of Autoimmune Disease, The Second Clinical Medical College of Jinan University, Shenzhen People’s Hospital, Shenzhen, Guangdong 518020, P.R. China.

***Corresponding authors:**

Yong Dai

E-mail address: daiyong22@aliyun.com

Donge Tang

E-mail address: donge66@126.com

Fanna Liu

E-mail address: tliufana@jnu.edu.cn

**Funding Information:**

This study was funded by the Fund of Guangxi Key Laboratory of Metabolic Diseases Research (grant number No.20–065-76).

Materials and Method

1. Material

Labeling kit and trypsin were obtained from Thermo and Promega (Fitchburg, WI), respectively, acetonitrile and ultrapure water were purchased from Fisher Chemical. Trifluoroacetic acid, formic acid, and protease inhibitors were provided by Sigma-Aldrich, Fluka (Buches, Germany), and Calbiochem, respectively. Iodoacetamide, dithiothreitol, urea, EDTA, NAM, TSA, triethylammonium bicarbonate were from Sigma (St. Louis, MO). BCA kits were obtained from Biyuntian (Shanghai, China).

1. Selection of research object and sample preparation

Under the guidance of the program approved by the Guangxi Key Laboratory of Metabolic Diseases Research Ethics Committee, we collected peripheral blood mononuclear cell samples from 8 SLE patients. All patients had no other obvious complications or serious primary diseases, such as cardiovascular disease, liver disease, etc. In addition, 8 normal controls were screened, and they did not have any other diseases through health examination. Each participant signed an informed consent form. After sample collection, PBMCs were isolated within 4 hours and then stored at -80°C.

1. Protein Extraction

Four volumes of lysis buffer (containing 8M urea, 1% protease inhibitors, 3 μM TSA, 50 mM NAM, and 2 mM EDTA) were added to the samples, respectively, followed by lysis with sonication and centrifugation (4 °C and 12,000 g, 10 minutes). After removing cell debris, transfer the supernatant to a new centrifuge tube. Finally, protein concentration determination was performed using BCA kit according to the manufacturer's instructions.

1. Trypsin digestion and TMT labeling

Before trypsinization, dithiothreitol was added to the prepared protein solution to a concentration of 5 mM. After reduction at 56 °C for 30 min, iodoacetamide was added to the protein solution，and its final concentration is 11 mM . Incubate for 15 min at room temperature in the dark, and finally dilute the protein solution to make the urea concentration below 2 M. Add trypsin (mass ratio of trypsin and protein 1:50) to the protein solution, and after enzymolysis overnight at room temperature, add trypsin again (mass ratio of trypsin and protein 1:100), and continue to enzyme at the same temperature solution for 4 h. fter digestion, the peptides were desalted using a Strata X C18 SPE column (Phenomenex, Torrance, CA), lyophilized in vacuo and reconstituted in 0.5 M TEAB. Finally, label the peptides according to the manufacturer's instructions of the TMT kit.

1. Antibody-based modifications for enrichment

The supernatant of the trypsin peptide dissolved in NETN buffer (100 mM NaCl, 1 mM EDTA, 50 mM Tris-HCl, 0.5% NP-40, pH 8.0) was transferred to a 2-hydroxyisobutyrylated resin (Lot number PTM-804, PTM Bio Inc, Hangzhou). Shake gently overnight on a rotating shaker at 4 °C. At the end of the incubation, wash four times with NETN buffer and twice with H2O. Afterwards, the bound peptides were fully eluted with an appropriate volume of 0.1% trifluoroacetic acid (TFA), and the eluate was collected and vacuum frozen and dried. Finally, the peptides were desalted with C18 ZipTips (Millipore), dried by vacuum spin dryer and then used for LC-MS/MS analysis.

The Anti-2-hydroxyisobutyryllysine antibody conjugated agarose beads used in this step are specific. By immobilization of highly specific anti-2-hydroxyisobutyryl lysine antibody, 2-hydroxyisobutyryl lysine antibody bead agarose selectively captures peptides/proteins bearing 2-hydroxyisobutyryl lysine residues, but does not cross-react with peptides/proteins bearing other structurally similar modified residues. As seen by the Dot blot results of Figure S3, only the 2-hydroxyisobutyrylated BSA showed spot signals, demonstrating the specificity of the above Anti-2-hydroxyisobutyryllysine antibody conjugated agarose beads.


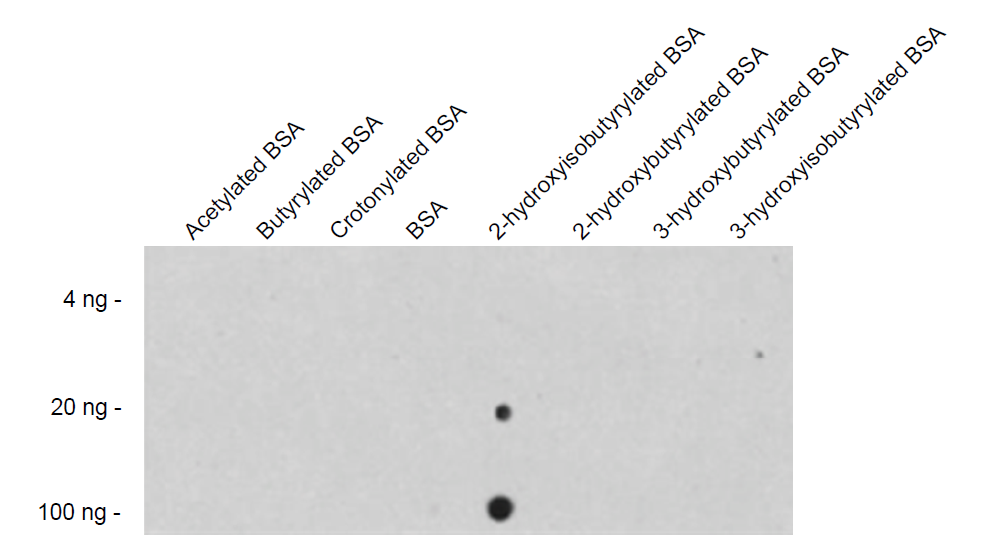


Figure S3 Dot blot analysis of anti-hydroxyisobutyryllysine antibody on different acylation proteins. This picture is from the instructions of PTM BIO's (www.ptm-biolab.com.cn)Anti-2-hydroxyisobutyryllysine antibody conjugated agarose beads (Lot number PTM-804).

1. LC–MS/MS analysis

Solvent A contained 0.1% formic acid and 2% acetonitrile, and solvent B contained 0.1% formic acid and 90% acetonitrile. The tryptic peptides were dissolved in solvent A and separated using the EASY-nLC 1000 ultra-high performance liquid phase system. The liquid phase gradient consisted of solvent B from 7% to 22% over 19 min, followed by an increase from 22% to 35% over 19–32 min, 80% over 32–36 min, and a final hold at 80% over 36–40 min. All the above steps were performed at a constant flow rate of 800 nL/min on the EASYnLC 1000 UPLC system. The peptides were separated and injected into the NSI ion source (voltage 2.0kV), and then connected to the UPLC device in the Q ExactiveTM Plus for MS/MS analysis. The scanning range of the primary mass spectrometer was set to 350-1800 m/z, and the scanning resolution was set to 70,000, which could comprehensively scan the peptide precursor ion and its secondary fragments. For peptides selected for MS/MS, the scan range was fixed at 100 m/z starting point and the Orbitrap scan resolution was set to 17,500. The data acquisition mode uses the Data Dependent Scanning (DDA) program. In order to improve the effective utilization of mass spectrometry, the automatic gain control (AGC) is set to 5E4, the signal threshold is set to 10000 ions/s, the maximum implantation time is set to 200 ms, and the dynamic exclusion time of tandem mass spectrometry scanning is set to 30 seconds to avoid repeated scanning of parent ions.

1. Database search

Maxquant (v1.5.2.8) was used for data analysis. Anti-libraries and common contamination libraries were added to the SwissProt Human (20130 sequences) database. Use Trypsin/P as the cleaving enzyme. The mass error tolerance of the primary precursor ions was set to 20 ppm and 5 ppm for the first search and the main search, respectively, and the mass error tolerance of the secondary fragments was 0.02 Da. Cysteine ​​alkylation was set as fixed modification, 2-hydroxyisobutylation of lysine, oxidation of methionine, and acetylation of protein N-terminus were set as variable modification.

1. MS quality control detection

Figure S1 shows the length distribution of the peptides identified by MS. Most of the peptides were distributed in the range of 7-20 amino acids, in accordance with the general pattern based on trypsin enzymatic digestion and HCD fragmentation mode. Peptides smaller than 5 amino acids do not yield valid sequence identification due to the small number of fragment ions generated. Peptides larger than 20 amino acids are not suitable for the HCD fragmentation method due to their high mass and charge number. The distribution of the peptide lengths identified by mass spectrometry was in accordance with the quality control requirements.


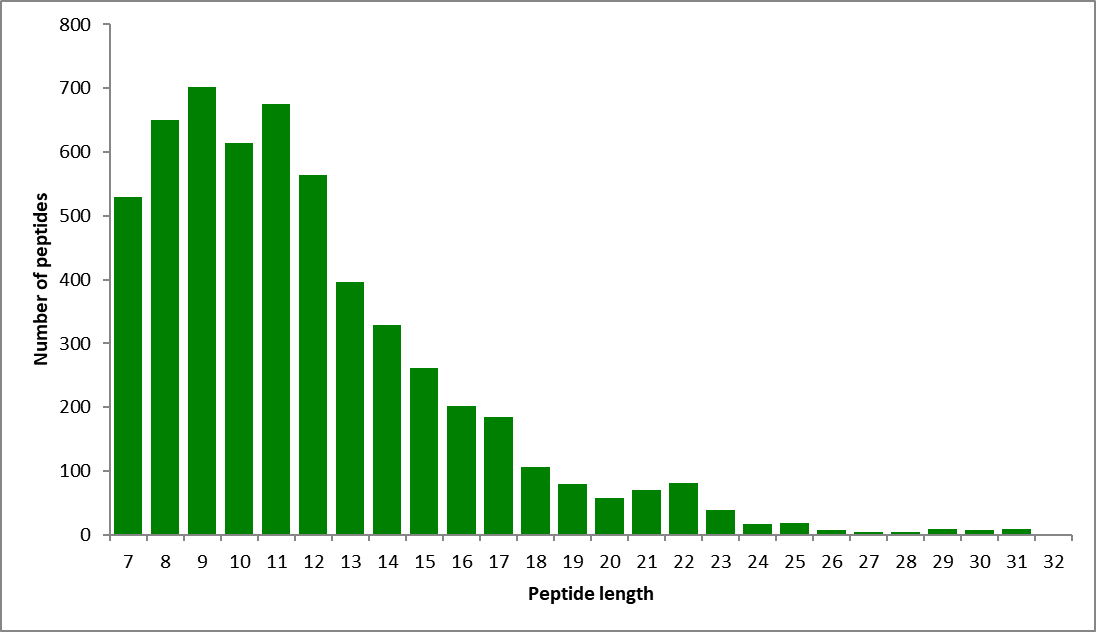


Figure S1 Length distribution of peptides identified by mass spectrometry.

Figure S2 shows the mass accuracy distribution of the mass spectrometry data. The first-order mass error of most of the spectra is within 10 ppm, which is in line with the high precision characteristics of orbital trap mass spectrometry. This indicates that the mass accuracy of the mass spectrometer is normal and does not affect the qualitative and quantitative analysis of the protein due to excessive mass deviation. The score of the spectra matching peptides (characterizing the confidence of peptide identification) is negatively correlated with the distribution of mass bias. The higher the score, the smaller the mass bias.

Figure S2 Mass accuracy distribution of mass spectrometry data.

1. Bioinformatic Method

For experimentally derived data, SLE/NC ratios ≥ 1.2 or ≤ 1/1.2 were considered differentially expressed. Wayne diagram is used to calculate the differential modification sites of overlapping up and down regulated Khib. Wolfpsort was used to annotate the subcellular localization of the protein. GO enrichment analysis is completed in David website (http://david.ncifcrf.gov). Then GO bubble maps were drawn on the Oebiotech website (https://cloud.oebiotech.cn/task/). KEGG analysis was performed by Cytoscape (v3.8.2) software. The original KEGG pathway map was downloaded from the Kyoto Encyclopedia of Genes and Genomes (KEGG) (https://www.genome.jp/kegg/). On the basis of the original pathway map, we use the materials from SERVER MEDICAL ART (https://smart.servier.com) to adjust and process the KEGG pathway map. The STRING website (https://cn.string-db.org） was used to analyze the interaction between proteins, and the Cytoscape (v3.8.2) software was used to draw the PPI network diagram. The MCODE plug-in in Cytoscape was used to extract the sub network, and the KEGG pathway enrichment analysis was carried out on the DMPs in the sub network.

Table S1 Characterization of expression and modification of key proteins in complement and coagulation pathways and platelet activation pathways

| Protein | Protein Expression | Protein Modification | Modification Site |
| --- | --- | --- | --- |
| A2M | Up | Up | K1176,K531,K567,K1019,K516 |
|  |  | Down | K314,K1003 |
| C3 |  | Up | K879,K1526,K155,K305,K1306 |
| C4B | Up | Down | K1658 |
| CFB | down | Down | K545,K707,K91 |
| F13A1 |  | Up | K74,K514,K584 |
|  |  | Down | K69 |
| F2 | Up | Down | K350 |
| FGA | down | Up | K558,K620,K71,K581 |
|  |  | Down | K157,K476,K575,K599,K463,K448 |
| FGB | down | Up | K353,K239,K328,K211 |
| FGG | down | Up | K84,K292,K196,K166,K231 |
| SERPINA1 | Up | Up | K411 |
|  |  | Down | K355,K160,K241,K178 |
| SERPING1 | Up | Down | K161,K385 |
| ACTG1 |  | Up | K328,K326 |
| FERMT3 | down | Up | K590,K262 |
|  |  | Down | K69,K222 |
| GNAI2 | down | Up | K318 |
| GNAQ |  | Up | K72 |
| GP5 | down | Up | K109 |
| ITGA2B |  | Down | K708 |
| ITGB1 |  | Down | K73 |
| ITGB3 |  | Up | K279,K380,K755 |
|  |  | Down | K644,K541,K436,K98 |
| LCP2 | down | Up | K438 |
| MAPK1 | down | Up | K138 |
| PPP1CC |  | Down | K41 |
| PTGS1 |  | Up | K565 |
| RAP1B | down | Up | K149,K104,K174,K151,K117 |
| RHOA | down | Up | K118,K7,K140 |
| ROCK1 |  | Up | K934 |
| ROCK2 | down | Up | K964,K901 |
| SRC |  | Up | K359,K354 |
| TLN1 | down | Up | K1332,K1933,K316,K1654,K2168,K2119, K268,K2104,K745,K164,K357 |
|  |  | Down | K2443,K1221,K2423,K149,K2445,K58,  K15,K137,K1190,K1522,K1947,K156,  K2274,K1170 |
| VASP | down | Up | K53 |
